# Protein biomarkers in pulmonary arterial hypertension: advances, clinical relevance, and translational challenges

**DOI:** 10.1186/s12967-025-07257-w

**Published:** 2025-11-14

**Authors:** Yanqin Niu, Jinglin Tian, Steeve Provencher, Sebastien Bonnet, Olivier Boucherat, François Potus, Deming Gou

**Affiliations:** 1 College of Life Sciences and Oceanography, Shenzhen University, Shenzhen, Guangdong China; 2 Pulmonary Hypertension Research Group, Québec Heart and Lung Institute Research Centre, Québec City, QC Canada; 3 Department of Medicine, Laval University, Québec City, QC Canada

**Keywords:** Pulmonary arterial hypertension (PAH), Biomarkers, Proteomics

## Abstract

Pulmonary arterial hypertension (PAH) is a progressive and life-threatening disease characterized by pulmonary vasoconstriction and right ventricular dysfunction. Although classical circulating biomarkers such as brain natriuretic peptide (BNP) and N-terminal proBNP (NT-proBNP) are widely used in clinical settings, their low specificity and substantial variability limit their diagnostic and prognostic accuracy. In recent years, emerging protein biomarkers, such as Apelin, Osteopontin, and Endostatin, have provided deeper insight into disease mechanisms but require further validation. The advent of high-throughput proteomic platforms, including SOMAscan, Olink, and mass spectrometry-based assays, has revolutionized biomarker discovery by enabling the identification of novel candidates with greater sensitivity and specificity. Several proteomics-discovered biomarkers, including LTBP-2, IGFBP family members, NET4, TSP2, and FGF-23, have demonstrated superior prognostic value and may complement or surpass current standards in risk stratification. In this review, we comprehensively examine the landscape of circulating protein biomarkers in PAH, compare key proteomic technologies, and highlight translational challenges such as assay standardization and cohort heterogeneity. We propose an integrative approach combining proteomic, imaging, and genomic data to enhance precision diagnostics and personalized treatment strategies for patients with PAH.

## Background

Pulmonary hypertension (PH) is a severe and progressive disease characterized by increased pulmonary arterial pressure (PAR), extensive vascular remodeling, and elevated hemodynamic burden on the right ventricle (RV), eventually leading to right heart failure and increased mortality [1]. Clinically, PH is defined by a mean pulmonary artery pressure (mPAP) greater than 20 mmHg at rest, confirmed by right heart catheterization [2, 3]. This disease is classified into five clinical categories: pulmonary arterial hypertension (PAH), PH associated with left heart disease, PH associated with lung diseases and/or hypoxia, PH associated with pulmonary artery obstructions, and PH with unclear and/or multifactorial mechanisms [2]. Of these, PAH is a rare, progressive disorder characterized by pulmonary vascular remodeling, resulting in high pulmonary artery pressure and progressive right ventricular dysfunction [4–7]. Recent registry data from economically developed countries indicate a PAH incidence and prevalence of approximately 6 and 48–55 cases per million adults, respectively [2]. Despite increased recognition of PAH in clinical practice in recent decades, survival rates remain relatively low, which are 92% at 1 year, 84% at 2 years, and 79% at 3 years [8].

**Fig. 1:**
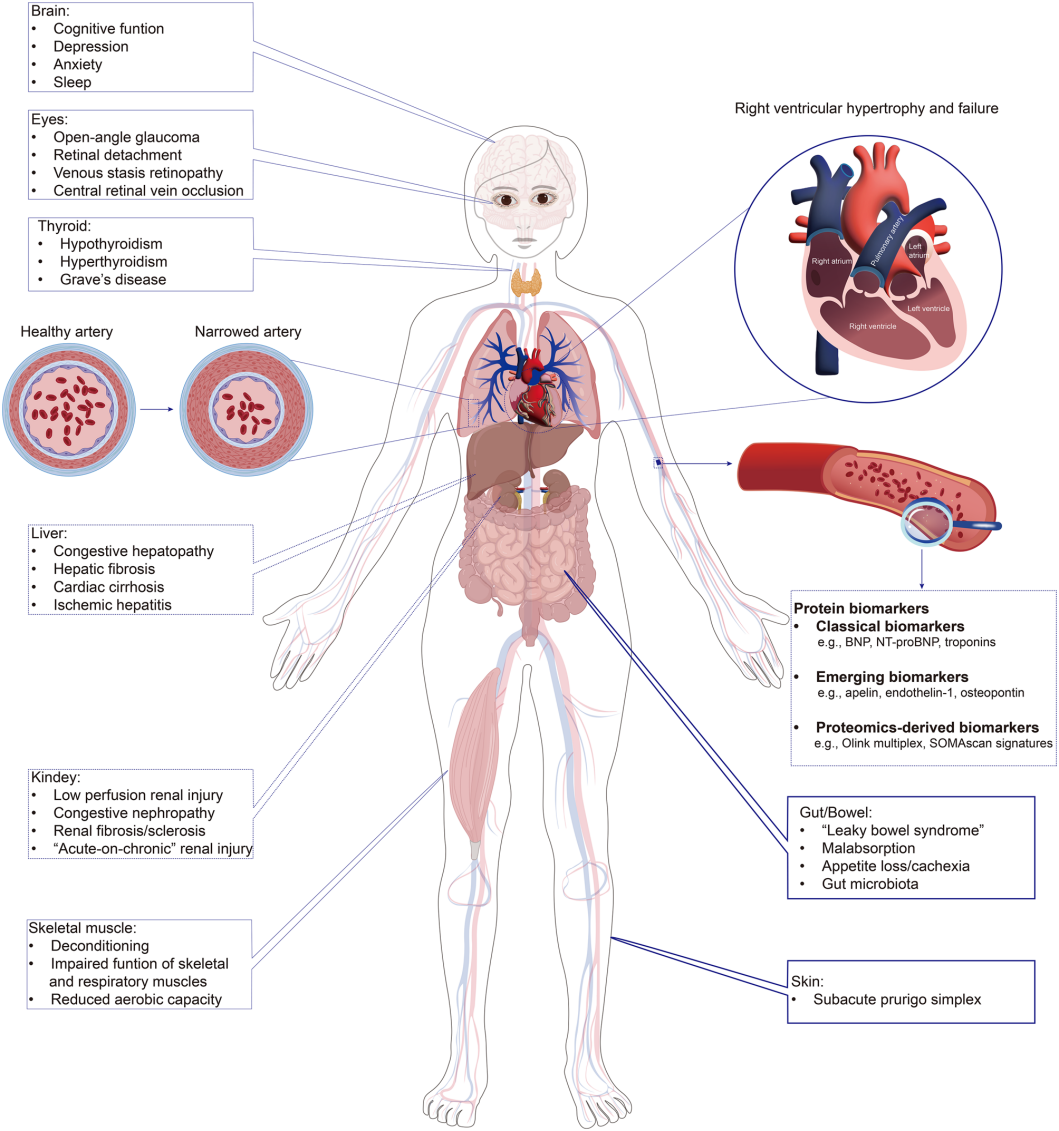
Systemic involvement of pulmonary arterial hypertension (PAH) and circulating protein biomarkers. This schematic illustrates PAH as a systemic disease that affects multiple organs beyond the cardiopulmonary system, including the brain, eyes, thyroid, liver, kidneys, gastrointestinal tract, skeletal muscle, and skin, in addition to right heart remodeling and pulmonary vascular changes. Such widespread involvement contributes to the nonspecific symptoms and frequent diagnostic delays observed in clinical practice. Circulating protein biomarkers, obtained from routine blood sampling, provide an accessible window into these processes. They can be broadly grouped into three categories: classical biomarkers (e.g., BNP, NT-proBNP, troponins), emerging biomarkers (e.g., apelin, endothelin-1, osteopontin), and proteomics-derived panels (e.g., Olink multiplex, SOMAscan signatures). Although detected in blood, these biomarkers reflect pathophysiological changes originating from different organs and systems, linking systemic manifestations to measurable molecular signatures. Representative examples are shown here, while a detailed, domain-based list is provided in Table 2

Traditionally, PAH has been predominantly recognized as a disease limited to the cardiopulmonary system. However, growing evidence indicates that PAH is better understood as a systemic disease, affecting multiple organ systems, including the left heart, brain, kidneys, liver, gastrointestinal tract, and skeletal muscle and others [9] (Fig. 1). Such extensive systemic involvement contributes to its clinical complexity, posing challenges for early and accurate diagnosis. Early clinical symptoms-such as exertional dyspnea, fatigue, chest discomfort, dizziness, and occasionally cough-are nonspecific and frequently lead to misdiagnosis or delayed diagnosis [2, 10]. Approximately 20% of patients experience significant diagnostic delays, often extending to 2–3 years and involving multiple consultations across different medical specialties [11]. Delayed initiation of targeted therapy significantly increases the risk of rapid disease progression, RV overload, and worse clinical outcomes, emphasizing the urgent clinical need for improved early diagnostic and risk stratification strategies [12].

Circulating biomarker testing offers advantages in population-based disease screening due to its non-invasive, cost-effective, and time-saving nature [13]. Blood analysis is the most widespread diagnostic procedure in medicine, with plasma and serum being readily accessible and commonly collected sample sources. Numerous studies have investigated the molecular mechanisms underlying PAH [14–17], identifying proteins, metabolites, and various forms of RNA as potential circulating biomarkers [18, 19]. Gene expression involves complex processes such as transcriptional regulation, alternative splicing, and RNA editing, with proteins executing these functions. Therefore, protein levels may provide an intermediate phenotype closer to the clinical phenotype of patients than raw transcriptome data [20, 21]. Proteomics, which studies the interactions, function, composition, and structures of proteins and their cellular activities [22], has made significant strides in discovering new biomarker candidates.

With the rapid development of multi-omics technologies and liquid biopsy approaches, numerous potential blood-based biomarkers have been identified. Among these, proteins such as brain natriuretic peptide (BNP), N-terminal pro-BNP (NT-proBNP), cardiac troponins I (cTnI) and T (cTnT) are key mediators of biological functions. Of which, the most established biomarkers in clinical PAH management, NT-proBNP and BNP, are proteins with well-recognized diagnostic and prognostic value. However, their expression levels are more strongly associated with cardiac function rather than PAH-specific pathological changes, showing significant variability [23]. This limits their utility in distinguishing between different PAH subtypes and performing accurate risk stratification. Advances in proteomics have significantly expanded the number of detectable proteins, leading to the identification of many novel protein biomarkers for PAH. With the increased use of unbiased detection methods, researchers can now identify a broader spectrum of biomarkers that may offer better specificity and sensitivity for PAH. Some of them may have demonstrated superior diagnostic and prognostic performance compared to NT-proBNP and BNP, showing promise for improving PAH management.

In this context, we define a biomarker as “a characteristic that is objectively measured and evaluated as an indicator of normal biological processes, pathogenic processes, or pharmacologic responses to a therapeutic intervention.” In contrast, a therapeutic target refers to a molecule that causally contributes to disease pathogenesis and can be modulated for therapeutic benefit. Notably, certain proteins in PAH, such as Endostatin or NEDD9, may serve dual roles as both biomarkers and therapeutic targets. In this review, we primarily focus on their value as circulating biomarkers, while acknowledging their potential therapeutic implications where relevant.

This review highlights recent progress in PAH biomarker research with a particular emphasis on novel proteomic methodologies and emerging protein biomarkers. We discuss the strengths and limitations of these technologies, critically evaluate the diagnostic and prognostic potential of newly discovered protein biomarkers, and outline remaining challenges and future directions for their clinical translation. Enhanced integration of these proteomic advances into clinical practice holds substantial promise for improving early detection, accurate prognosis, and individualized treatment in PAH patients, ultimately aiming to improve patient survival and quality of life.

## Maintext

To provide a structured overview of how proteomics informs biomarker development in PAH, this review is organized into three major sections: (i) advances in proteomic technologies, (ii) classification and clinical applications of circulating protein biomarkers, and (iii) current challenges and future directions for clinical translation.

### Advances in proteomic technologies and its contribution to biomarker discovery

**Fig. 2:**
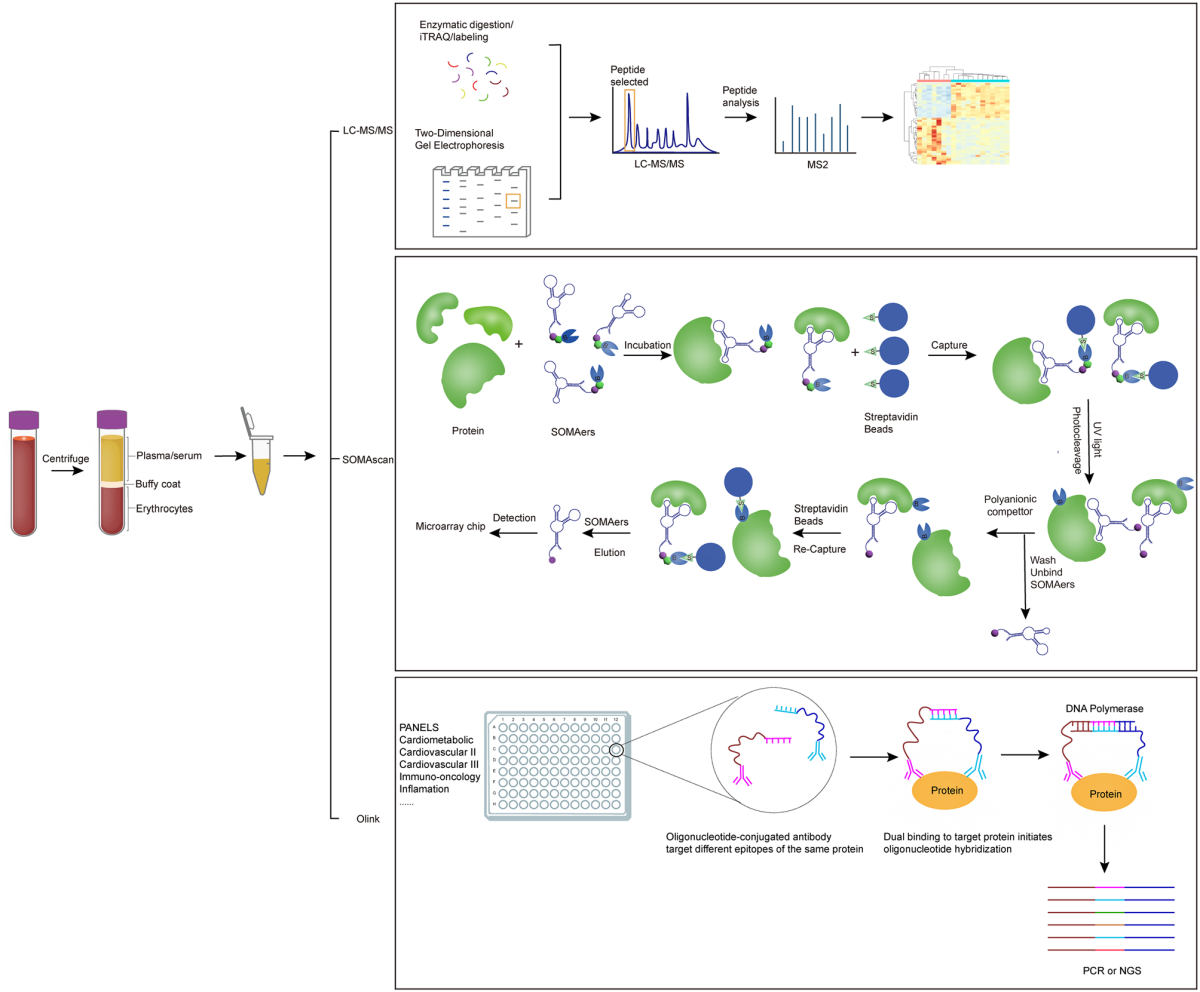
Main proteomic technologies in biomarker discovery for pulmonary arterial hypertension (PAH). This figure summarizes representative proteomic platforms applied to circulating biomarker discovery in PAH. Traditional MS-based approaches, such as liquid chromatography-mass spectrometry (LC-MS) and examples including two-dimensional gel electrophoresis (2-DE) coupled with MS and isobaric tagging techniques such as iTRAQ, provide high-resolution protein identification and quantification. However, these workflows are limited by complexity, labor-intensive preparation, reproducibility issues, and high costs, restricting their scalability in large clinical studies. Affinity-based technologies, including Olink proximity extension assay and SOMAscan aptamer-based assay, overcome many of these challenges by enabling highly multiplexed, sensitive, and reproducible measurement of hundreds to thousands of proteins from minimal plasma or serum volumes. These advances have greatly expanded proteome coverage and accelerated the identification of both individual and multi-protein biomarker signatures with diagnostic and prognostic potential in PAH

Proteomic research enables the identification of whole protein profiles and constitutes a powerful method for discovering novel biomarkers [21]. Over the past decade, significant technical advances in high-throughput proteomic technology have greatly influenced PAH biomarker discovery, enabling sensitive and reproducible qualitative and quantitative measurement of thousands of analytes simultaneously in biological fluids (Fig. 2).

Traditional methods, such as liquid chromatography-mass spectrometry (LC-MS), have long been indispensable in proteomics [24]. LC-MS typically employs a bottom-up approach, in which proteins are enzymatically digested into peptides, separated chromatographically, and analyzed via mass spectrometry [25]. High-performance liquid chromatography (HPLC) further enhances this method by enabling continuous separation of proteins, and when combined with MS, it allows for higher throughput [26]. Other MS-based techniques, such as two-dimensional gel electrophoresis (2-DE) and isobaric tagging techniques like iTRAQ, have also been widely used for protein separation and quantification [27, 28]. Despite providing high resolution and accuracy, these methods are hindered by their complexity, reliance on extensive sample preparation, poor reproducibility, and high costs. These limitations, including challenges in scalability and consistency across studies, restrict their widespread application, particularly in large cohort studies.

Recent breakthroughs in affinity-based protein profiling technologies, such as the Olink and SOMAscan platforms, have revolutionized proteomic research by addressing many of the limitations of traditional methods. The Olink proximity extension assay utilizes a dual recognition system, where two different epitope-directed antibodies are conjugated to partially complementary barcodes. Upon binding to the target protein, the oligonucleotides brought into proximity undergo hybridization to form a double strand, which is then amplified by PCR and analyzed by qPCR or next-generation sequencing [29]. This method offers high specificity and sensitivity, allowing for the detection of low-abundance proteins even in small sample volumes. Furthermore, the Olink platform’s compatibility with high-throughput workflows makes it ideal for identifying novel biomarkers in PAH.

The SOMAscan assay, on the other hand, uses selective, slow off-rate modified aptamers (SOMAmers) to bind specific proteins. Capable of quantifying up to 7000 proteins per biosample, SOMAscan provides comprehensive proteome coverage [30]. When targeting fewer analytes, it offers high precision and dynamic range, enabling the quantification of proteins that may not be measurable by other platforms. These technologies have significantly accelerated biomarker discovery by providing broader proteome coverage, better reproducibility, and compatibility with high-throughput analysis (Table 1).

**Table 1:** Comparison of proteomic technologies for biomarker discovery in PAH

Feature	LC-MS (Liquid chromatography-mass spectrometry)	Olink (Antibody-based proximity extension assay)	SOMAscan (Aptamer-based assay)
Technology principle	Mass spectrometry-based, peptide separation and identification	Dual antibody recognition with DNA barcode amplification	Aptamer binding with slow off-rate modified nucleic acids (SOMAmers)
Detection type	Untargeted & targeted	Targeted	Targeted
Sensitivity	High (variable for low-abundance proteins)	Very high	Very high for medium/high-abundance proteins; lower for low-abundance proteins compared to Olink.
Specificity	High	Very high (antibody dependent)	High (aptamer dependent)
Sample preparation	Complex	Simple	Simple
Throughput capability	Medium (dozens to hundreds of proteins)	High (dozens to hundreds per panel; up to thousands with multiple panels)	Very high (up to ~ 7000 proteins)
Reproducibility	Variable (influenced by sample preparation)	High	High
Cost	High (equipment-intensive)	Moderate to high	High
Advantages	Accurate quantification, novel protein identification	High sensitivity, specificity, minimal sample use	Broad proteome coverage, very high multiplexing capability
Limitations	Complexity, lower throughput	Dependent on antibody availability	Dependent on aptamer availability

### Circulating protein biomarkers in PAH: diagnostic and prognostic applications

PAH remains a clinical challenge due to its insidious onset, complex pathobiology, and limited availability of non-invasive diagnostic tools. While right heart catheterization remains the gold standard for diagnosis, circulating protein biomarkers have emerged as valuable tools to support early detection, risk stratification, and prognosis evaluation in PAH. Protein biomarkers, accessible through blood sampling, can reflect diverse pathological processes such as myocardial strain, vascular remodeling, inflammation, and end-organ damage. To reflect their progression from clinical application to experimental discovery, circulating protein biomarkers are grouped into classical, emerging, and proteomics-derived categories, providing a systematic framework for understanding their roles in PAH diagnosis and management (Table 2).

**Table 2:** Overview of Circulating protein biomarkers in PAH, categorized by clinical maturity and discovery platform

Category	Biomarker(s)	Pathophysiological domain (with mechanistic context) / platform	Biomarker type + Clinical relevance	Reference
Classical	BNP, NT-proBNP	**Cardiac Function/Damage**: released by RV under wall stress	**Diagnostic + Prognostic**; correlate with hemodynamics, severity, prognosis; incorporated in guidelines; limited specificity	[2, 31–39]
	cTnI, cTnT (Troponins)	**Cardiac Function/Damage**: markers of cardiomyocyte injury	**Diagnostic + Prognostic**; optional diagnosing marker in guide; predict adverse outcomes; limited specificity	[41–42]
Emerging	Apelin	**Cardiac Function/Damage**: vascular development, contractility	**Diagnostic**; sensitive IPAH biomarker; higher AUC vs. NT-proBNP	[43–47]
	Osteopontin	**Cardiac Function/Damage**: secreted by cardiomyocytes and fibroblasts	**Diagnostic**; elevated in IPAH, CHD-PAH, CTD-PAH; correlates with mPAP, RAP, CI, PVR	[48–51]
	Endostatin	**Cardiac Function/Damage**: angiostatic peptide from COL18A1	**Diagnostic + Prognostic**; correlates with RV function, hemodynamics, NT-proBNP, 6MWD	[52, 53]
	Endothelin-1 (ET-1)	**Vascular Remodeling/Endothelial Dysfunction**: potent smooth muscle mitogen	**Diagnostic + Prognostic**; elevated in PAH; correlates with PVR, mPAP, CO, CI,6MWD; responsive to therapy	[54–58]
	Copeptin	**Vascular Remodeling/Endothelial Dysfunction**: surrogate of AVP release	**Diagnostic + Prognostic**; elevated in PAH; correlates with NYHA class, 6MWD, NT-proBNP, renal function; decreases with therapy; predicts mortality; relates to mPAP, PVR, pulmonary blood flow in CHD-PAH	[59–62]
	Activin A, FSTL3	**Vascular Remodeling/Endothelial Dysfunction**: Smad2/3 pathway activation	**Diagnostic + Prognostic**; elevated in PAH; independently predict death or transplantation	[63, 64]
	HDGF	**Vascular Remodeling/Endothelial Dysfunction**: growth factor secretion	**Diagnostic + Prognostic**; elevated in PAH; correlates with WHO FC, mortality; diagnostic value but levels also raised in Down syndrome	[65–67]
	CTHRC1	**Fibrosis**: secreted ECM protein	**Prognostic**; correlates with RV dysfunction; levels decrease after BPA	[68, 69]
	sST2	**Fibrosis**: IL-1 receptor family; reflects myocardial fibrosis and remodeling	**Diagnostic + Prognostic**; correlates with CI, PVR, WHO FC, RV volumes/function; predicts outcomes	[70–72]
	Galectin-3	**Fibrosis**: mediates TGF-β1-driven cardiac fibrosis	**Diagnostic + Prognostic**; elevated in IPAH and CTD-PAH; correlates with severity	[73–75]
	NEDD9	**Fibrosis**: regulates COL3A1 via NKX2-5	**Diagnostic + Prognostic**; elevated in PAH plasma; predicts diagnosis and prognosis	[76]
	Cytokines (IL-1β, -2, -4, -6, -8, -10, -12p70, TNFα)	**Inflammation/Oxidative Stress**: inflammatory mediators	**Diagnostic + Prognostic**; elevated in PAH; IL-6, IL-8, IL-10, IL-12p70 predict survival	[77]
	VEGF, PDGF, TGFβ1	**Inflammation/Oxidative Stress**: growth factor signaling	**Diagnostic**; elevated in PAH; correlate with CI, PVR, SvO₂	[78, 79]
	Ang-2	**Vascular Remodeling/Endothelial Dysfunction**: vascular growth factor	**Diagnostic + Prognostic**; elevated in IPAH; independent predictor of mortality	[79]
	GDF-15	**Inflammation/Oxidative Stress**: TGF-β superfamily cytokine	**Prognostic**; correlates with right atrial/wedge pressure, SvO₂,NT-proBNP, uric acid	[80]
	CTGF	**Inflammation/Oxidative Stress**: pro-fibrotic growth factor	**Diagnostic + Prognostic**; correlates with PASP, mPAP, PVR, SvO₂; comparable to BNP in IPAH	[81]
	CRP	**Inflammation/Oxidative Stress**: acute phase reactant	**Diagnostic + Prognostic**; elevated in PAH; correlates with NYHA class, RAP, 6MWD; predicts mortality	[82]
	Creatinine	**End-organ Dysfunction**: renal function marker	**Diagnostic + Prognostic**; elevated in PAH; correlates with RAP, CI; predicts mortality	[83–85]
	Cystatin C	**End-organ Dysfunction**: novel renal biomarker linked to RV remodeling	**Diagnostic + Prognostic**; elevated in PAH; correlates with RV mass, function, strain; inversely with RVEF and e′ velocity	[86]
Proteomics-derived	LTBP-2, COL18A1, COL6A3, TNC, CA1	Olink multiplex (RV tissue + plasma in PAH with RV decompensation)	**Prognostic**; elevated in RV tissue/plasma; LTBP-2 outperforms NT-proBNP	[87]
	CA2, KLKB1, IGFBP1-7	DDA-MS (Orbitrap) on plasma (IPAH vs. healthy controls)	**Prognostic**; correlate with PVR, 6MWD, survival	[88–90]
	9-protein panel (IL1R4, EPO, Factor D, IGFBP1, TIMP1, TIMP2, Factor H, Plasminogen and EPO)	SOMAscan V3 (1129 plasma proteins across PAH cohorts)	**Prognostic**; independently predicted survival beyond NT-proBNP	[91]
	6-marker panel (SVEP1, PXDN, renin, NRP1, TSP2, PRDX4)	SOMAscan V4 (> 4000 proteins across PAH cohorts)	**Prognostic**; improved risk stratification with clinical variables	[92]
	NET4, TSP2	SOMAscan V4 (4152 proteins in idiopathic & heritable PAH vs. controls; pQTL & MR)	**Prognostic**; NET4 predicts PAH risk & poor survival; TSP2 elevation is protective	[93]
	FGF-23	Olink metabolic panel (PAH at diagnosis & early follow-up vs. healthy controls)	**Diagnostic + Prognostic**; levated in PAH; correlates with disease severity and hemodynamics; decreases with therapy; potential disease monitoring marker	[94]
	AXIN-1, ST1A1, CXCL1, STAMBP, SIRT2, and CASP8	Olink inflammation panel (IPAH vs. SSc-PAH plasma)	**Diagnostic**; elevated in IPAH but not SSc-PAH	[95]
	VEGFA, CD8A, CXCL9, MMP1, IL-15RA, PD-L1, HGF, TNF, X4EBP1, CCL28, and CD40		**Diagnostic**; elevated in IPAH and SSc-PAH	
	FSTL3, SPON1	Olink inflammation panel (treatment-naïve PAH vs. controls)	**Diagnostic + Prognostic**; predict death or transplant; add value beyond risk scores	[63, 92, 96]
	C4a des Arg	SELDI-TOF MS(plasma IPAH vs. controls)	**Diagnostic + Prognostic**; elevated in IPAH; 92% classification accuracy	[97]
	LRG (Leucine-rich α-2-glycoprotein)	2-DE + MALDI-TOF (serum IPAH vs. normals)	**Diagnostic + Prognostic**; elevated in IPAH; correlates with RV diameters	[98]
	ADIPO, DBH, ANPEP	iTRAQ proteomics (plasma in pediatric VSD with vs. without PAH)	**Diagnostic + Prognostic**; composite risk score (AUC 0.87) predicts PAH onset	[99]

#### Classical biomarkers: BNP, NT-proBNP and troponins

Among protein biomarkers, **BNP** and its inactive fragment, **NT-proBNP** are the most established circulating biomarkers in PAH and remain the only ones routinely incorporated into clinical guidelines [2]. Both are released by cardiomyocytes in response to ventricular wall stress, volume overload, and increased afterload—hallmarks of RV strain in PAH [31, 32]. Their plasma concentrations correlate with pulmonary hemodynamics, right atrial pressure (RAP), RV size, and cardiac output (CO), and they reliably reflect disease severity and prognosis [33–36].

Clinically, BNP and NT-proBNP are central to both initial risk assessment and longitudinal monitoring in PAH. Current ESC/ERS guidelines support their use for prognostic evaluation, and both peptides have been incorporated into several validated risk stratification models [2]. A four-strata risk assessment strategy has proposed specific cut-off values for BNP and NT-proBNP to define low, intermediate-low, intermediate-high, and high-risk groups: BNP < 50, 50–199, 200–800, and > 800 ng/L, and NT-proBNP < 300, 300–649, 650–1100, and > 1100 ng/L, respectively [37]. In the REVEAL registry, BNP and NT-proBNP levels were strongly associated with long-term survival. Patients with BNP < 50 pg/mL or NT-proBNP < 300 pg/mL demonstrated significantly better outcomes, while those with BNP > 180 pg/mL or NT-proBNP > 1500 pg/mL had a higher risk of morbidity and mortality [38].These thresholds were further incorporated into the REVEAL 2.0 risk score, a widely used tool to predict 1-year survival in PAH. The total score, ranging from 1 to 15, stratifies patients into five survival categories, with ≥ 95% 1-year survival at the low-risk end and < 70% at the high-risk end [39].

Furthermore, reductions in NT-proBNP levels after initiating PAH-targeted therapies are associated with improved exercise capacity, hemodynamics, and survival, underscoring its value as a surrogate treatment response marker. Conversely, persistently elevated levels portend poor outcomes and may indicate the need for therapeutic escalation. These peptides also have prognostic utility in PAH, though baseline values may differ depending on comorbidities such as renal impairment or left heart dysfunction [34–36, 38].

Despite their utility, BNP and NT-proBNP are not specific to PAH and can be elevated in other cardiac or systemic conditions. Their interpretation must therefore be contextualized within initial evaluation of symptoms, family history, and physical examination. Nonetheless, due to their strong reproducibility, wide availability, and validated prognostic value, BNP and NT-proBNP remain the cornerstone of biomarker-based PAH risk assessment in routine clinical practice.

**Cardiac troponins (cTnI**,** cTnT)** are essential proteins in cardiac muscle and are highly sensitive markers for detecting myocardial damage, revolutionizing the understanding of minor myocardial injury and infarction [40]. Troponin is recommended as an optional test for diagnosing PAH when clinically appropriate in 2022 ESC/ERS Guidelines [2]. However, these markers are not universally detectable in PAH patients. Heresi et al. reported that cTnI was detectable in 25% of PAH patients with a lower limit of detection of 0.008 ng/mL, whereas none of the controls showed detectable levels of cTnI [41]. Torbicki et al. found that cTnT was detectable in 7 out of 51 (14%) PAH patients. Compared to those without detectable cTnT, patients with detectable levels had higher serum NT-proBNP levels and covered less distance during the 6-Minute Walk Distance (6MWD) test [42]. Similarly, the use of troponins in PAH is limited by their lack of specificity; elevated levels can also occur in conditions such as acute coronary syndromes, heart failure, cardiomyopathies, myocarditis, renal failure, tachyarrhythmias, pulmonary embolism, and even after strenuous exercise in healthy individuals [100].

#### Emerging biomarkers associated with distinct pathophysiological processes

Emerging biomarkers have been identified that reflect diverse pathophysiological mechanisms in PAH, such as myocardial remodeling, endothelial dysfunction, inflammation, and extracellular matrix (ECM) fibrosis, providing insights beyond what classical markers can offer.

##### Cardiac function/damage

The peptide **Apelin** and the **Apelin receptor** are present in the heart, as well as in the systemic and pulmonary vasculature. They are involved in vascular development, regulation of apoptosis, NO-dependent vasodilation, and improvement of cardiac contractility [43]. Circulating Apelin has emerged as a potential biomarker for pre-capillary PH (including PAH and other PH) with a high sensitivity and specificity [44]. Among apelin fragments, Apelin-17 appears to be a suitable blood-derived diagnostic marker for idiopathic PAH (IPAH), exhibiting a higher AUC compared to NT-proBNP [45]. Suparna et al. found lower serum concentrations of Apelin-12 in patients with PAH compared to controls [46]. Furthermore, plasma concentrations of Apelin-36 were observed to significantly decrease in patients with IPAH, but also with a decrease in those with chronic parenchymal lung disease and chronic heart failure [47].

**Osteopontin** is a glycoprotein expressed in both cardiomyocytes and fibroblasts, upregulated in individuals with heart failure, and may thus represent a new biomarker that facilitates risk stratification in patients with heart failure [48]. Elevated circulating Osteopontin levels have been reported in patients with various forms of PAH, including IPAH [49], congenital heart disease-associated PAH (CHD-PAH) [50] and connective tissue disease-associated PAH (CTD-PAH) [51]. Studies have demonstrated a correlation between circulating Osteopontin levels and hemodynamic parameters such as mPAP [50], RAP [49], cardiac index (CI) [50], and total pulmonary vascular resistance (PVR) [50] in patients with PAH.

**Endostatin**, an angiostatic peptide derived from the carboxy terminus of the extracellular matrix protein collagen XVIII (Col18a1), is elevated in PAH, and a strong correlation has been observed between circulating Endostatin levels and markers of RV structure and function [53]. Notably, a loss-of-function variant in the Col18a1 gene, which encodes Endostatin, has been linked to reduced circulating Endostatin levels and independently associated with improved mortality outcomes. Additionally, serum Endostatin levels have shown significant correlations with hemodynamics, NT-proBNP, and 6MWD, further supporting its potential as a biomarker for disease progression and poor prognosis in PAH. Importantly, these findings highlight Col18a1/ Endostatin as a potential new therapeutic target, opening up avenues for targeted treatment strategies in PAH [53].

##### Vascular remodeling/endothelial dysfunction

**Endothelin-1 (ET-1)** acts as a direct smooth muscle cell mitogen by activating both ETA- and ETB-receptors and stimulates the production of cytokines and growth factors [54]. Plasma ET-1 levels are significantly elevated in patients with PAH [55]. A strong correlation was observed between PVR, mPAP, CO, CI, and 6MWD [56]. Following 6 months of bosentan treatment, there is a significant increase in 6MWD and improvement in World Health Organization Functional Classification (WHO FC), as well as a significant decrease in BNP levels and a trend toward lower ET-1 plasma levels [57]. Elevated ET-1 plasma levels independently predict long-term clinical worsening [58]. However, ET-1 plasma levels do not accurately reflect tissue concentration and are influenced by demographic factors and medications, potentially confounding results. ET receptor antagonists used in PH treatment can limit ET-1’s use as a biomarker due to difficulty distinguishing between disease- and treatment-related effects [101].

**Copeptin**, the C-terminal segment of the arginine vasopressin (AVP) precursor peptide, serves as a sensitive and stable surrogate marker for AVP release [59]. AVP induces endothelium-dependent vasodilatation [60]. Circulating Copeptin levels were found to be higher in PAH patients compared to diseased controls. Baseline Copeptin levels were correlated with New York Heart Association (NYHA) functional class, 6MWD, NT-proBNP, Creatinine, and estimated glomerular filtration rate. However, Copeptin levels did not show a correlation with hemodynamics but decreased following the initiation of PAH therapy. Elevated Copeptin levels were associated with shorter survival and were identified as independent predictors of mortality [61]. Plasma Copeptin levels were also observed to be elevated in children with CHD-PAH. Additionally, a statistically significant positive correlation was found between plasma Copeptin levels and and mPAP, PVR, and pulmonary blood flow. Conversely, there was a statistically significant negative correlation between plasma copeptin levels and right ventricular diastolic function [62].

**Activin A**, a dimer of the inhibin-βA subunit encoded by INHBA, is implicated in Smad2/3-pathway overactivation and the progression of obstructive vascular remodeling in PAH [102]. The activity of Activin A is regulated by endogenous inhibitors, including **Follistatin** and **Follistatin-like 3 (FSTL3)** [63]. Elevated circulating levels of Activin A and follistatin have been reported in PAH [64]. Guignabert et al. also demonstrated that increased serum levels of Activin A and FSTL3 predict death or lung transplantation in both a discovery cohort and an independent external PAH cohort, highlighting their potential as prognostic biomarkers in PAH [63].

**Hepatoma-derived growth factor (HDGF)** is a secreted multifunctional protein that may play an active role in vascular remodeling [65]. Yang et al. found that serum HDGF levels were significantly elevated in two independent PAH cohorts. Elevated HDGF was associated with worse WHO FC, exertional intolerance, and increased mortality in PAH, suggesting that HDGF could serve as a potential biomarker for predicting mortality and may have diagnostic value in distinguishing PAH from non-PAH conditions [66]. Six years later, Yang et al. confirmed that higher serum HDGF levels were linked to increased mortality and were associated with disease severity in a large multi-center adult PAH cohort (*n* = 2017) [65]. However, Yang and colleagues later found that Endostatin, Galectin-3, HDGF, and ST2 were also elevated in subjects with Down syndrome, regardless of PH status. NT-proBNP and IL-6 levels were similar between the Down syndrome with PH group and the non-Down syndrome PH group [67]. Therefore, while HDGF is useful for improving the diagnosis and prognosis of PH, as demonstrated here, its levels can be altered in genetically distinct populations such as individuals with Down syndrome. This highlights the importance of evaluating clinical biomarkers in specific groups and developing population-specific nomograms for more accurate assessment.

##### Fibrosis

PAH is a condition characterized by pathological changes in the ECM deposition in both distal pulmonary arteries and the RV [103, 104]. While a healthy ECM plays a crucial role in maintaining tissue structure and preventing dilation under pressure overload, increased fibrosis thickens vessel walls and leads to lumen occlusion, causing a loss of elasticity and vessel stiffening. Also, ECM remodeling and fibrosis in the RV contribute to impaired cardiac function. This pathological ECM accumulation and crosslinking, which stiffens both the vessels and the RV, presents potential therapeutic targets for PAH.

Several biomarkers are being explored for their role in monitoring RV dysfunction and fibrosis in PAH. Yokokawa and colleagues, investigate the potential of **collagen triple helix repeat-containing protein 1 (CTHRC1**), a secreted glycoprotein, as a novel biomarker for right ventricular dysfunction and fibrosis. Elevated plasma levels of CTHRC1 correlate with clinical indicators of right ventricular failure, and its levels decrease following successful balloon pulmonary angioplasty (BPA) in chronic thromboembolic pulmonary hypertension (CTEPH), suggesting its role in monitoring therapeutic efficacy [68, 69].

Another promising biomarker is **soluble ST2 (sST2)**, a member of the interleukin-1 (IL-1) receptor family, which has been identified as a potential marker for myocardial fibrosis and remodeling in various cardiovascular diseases [70]. Zheng et al. found that sST2 levels were significantly elevated in patients with IPAH. These elevated plasma sST2 levels correlated with the CI and PVR, indicating a possible reflection of PAH disease severity. Additionally, elevated sST2 levels were associated with poor clinical outcomes, suggesting its potential utility in predicting prognosis in PAH patients [71]. Carlomagno et al. also confirmed that levels of sST2 in serum of PAH patients were significantly higher. Additionally, patients with high sST2 showed significantly worse WHO FC, right ventricular volumes and systolic function [72].

**Galectin-3** has been reported to mediate the TGF-β1-induced cardiac fibrotic process [73]. A pilot study assessing the relationship between plasma Galectin-3 levels and validated risk scores in PAH patients found that Galectin-3 levels increased progressively with higher risk strata according to the REVEAL 2.0 risk score. Additionally, Galectin-3 levels were significantly lower in low-risk patients as defined by the ESC/ERS guidelines and echocardiographic evaluation of right heart performance, indicating its potential role in risk stratification [74]. Another study further supported Galectin-3 as a potential biomarker, showing elevated levels of Galectin-3 and aldosterone in patients with IPAH and CTD-PAH, suggesting their relevance in assessing disease severity and progression [75].

**NEDD9** plays a crucial role in the regulation of pulmonary endothelial fibrosis by transcriptionally upregulating COL3A1 through NKX2-5, which promotes collagen III synthesis and contributes to the development of PH. Genetic depletion or siRNA knockdown of NEDD9 has been shown to inhibit pulmonary vascular fibrosis and alleviate experimental PH [105]. Furthermore, Samokhin et al. found that NEDD9 levels were significantly elevated in the plasma of PAH patients, with plasma NEDD9 levels serving as a strong predictor of disease diagnosis. These findings suggest that NEDD9 could be a valuable diagnostic and prognostic biomarker for PAH [76].

##### Inflammation/oxidative stress

Inflammation is a driver of PAH, and increased cytokines and chemokines in the peripheral blood of patients with PAH correlate with survival and disease severity [106]. Several **cytokines**, including **IL -1β**,** -2**,** -4**,** -6**,** -8**,** -10**, and **− 12p70**, along with **tumor necrosis factor-α (TNFα)**, were elevated in PAH. Moreover, IL-6, 8, 10, and 12p70 levels were found to predict patient survival [77]. These results align with Selimovic et al.‘s findings, which reported significantly higher levels of IL-6, **TGFβ1**,** PDGF**, and **VEGF** in PAH patients versus controls. Additionally, a significant association between IL-6 and mortality was observed [78]. Plasma VEGF levels correlated with CI, PVR, and mixed venous oxygen saturation (SvO_2_) in IPAH [79]. **Angiopoietin-2 (Ang-2)** levels were also elevated in IPAH patients compared to healthy individuals and disease controls and correlated with various hemodynamic parameters. Elevated Ang-2 levels were identified as an independent risk factor for mortality [79]. **Growth differentiation factor-15 (GDF-15)**, a member of the TGFβ superfamily, was associated with increased mean right atrial and pulmonary capillary wedge pressures, a lower SvO_2_, and higher levels of uric acid and NT-proBNP in IPAH [80]. Plasma concentrations of **connective tissue growth factor (CTGF)** positively correlated with pulmonary artery systolic pressure (RVSP), mPAP, and PVR, and inversely correlated with SvO_2_. The diagnostic efficacy of CTGF in PAH was comparable to that of BNP [81].

**C-reactive protein (CRP)** is a marker of inflammation and tissue damage. Circulating CRP levels were found to be elevated in patients with PAH compared to those in control subjects. CRP levels correlated with NYHA functional class, RAP and 6MWD, and they were significantly higher in nonsurvivors than in survivors [107]. Besides, Giancarlo et al. observed that lower serum CRP levels were associated with higher 6MWD and improved survival rates of PAH patients [82].

##### End-organ dysfunction

Renal dysfunction is associated with a worse hemodynamic profile and acts as an independent predictor of mortality in PAH [83]. Serum **creatinine (SCr)** provides a basic evaluation of renal function, and it is readily available in clinical settings [84]. In a cohort study by Sanjiv et al., involving 500 patients with PAH, elevated SCr levels were found to be associated with higher RAP, lower CI, and increased mortality rates [83]. Rhodes et al. found that creatinine is correlated with RAP and CI, indicating disease severity, and may be utilized to predict survival in patients with IPAH [85].

**Cystatin C (CysC)** a novel marker of renal function, predicts left heart failure and cardiovascular mortality. Fenster et al. found that CysC was abnormally elevated in the PAH cohort when compared with controls. CysC positively correlated with RV end-diastolic volume, RV end-systolic volume, mass index, strain and strain rate and negatively correlated with RVEF and tricuspid valve e’ velocity [86].

#### Novel biomarkers identified through proteomic discovery

In addition to established diagnostic and prognostic markers, high-throughput proteomic technologies have opened a new frontier for identifying novel circulating protein biomarkers in PAH. Platforms such as SOMAscan, Olink, and data-dependent acquisition mass spectrometry (DDA-MS) now enable unbiased quantification of thousands of proteins from small-volume plasma samples with high sensitivity, reproducibility, and minimal sample processing.

For instance, Boucherat and colleagues employed a multi-omics approach integrating RV transcriptomic and proteomic profiling with cardiometabolic plasma protein analysis using the Olink platform. They identified five proteins (**COL18A1**,** LTBP-2**,** TNC**,** COL6A3**, and **CA1)** consistently elevated in RV tissue and plasma of PAH patients with RV decompensation. Among these, LTBP-2 showed the highest AUC, significantly outperforming NT-proBNP. The authors also demonstrated that LTBP-2 correlated with RV function and added incremental value to current risk stratification models for predicting survival in two PAH cohorts [87].

In another study, Nies et al. applied DDA-MS on trypsin-digested plasma samples using an Orbitrap platform, identifying 153 plasma proteins differentially expressed between IPAH patients and healthy controls. Among them, **carbonic anhydrase 2 (CA2)**, **kallikrein (KLKB1)**, and members of the insulin-like growth factor-binding protein (**IGFBP**) family (**IGFBP1-7**) were validated by immunoassay. Notably, IGFBP1 and IGFBP2 correlated with elevated PVR, and IGFBP2, 4, and 7 with reduced 6MWD and survival [88]. In a separate effort, Torres et al. specifically investigated serum IGFBP4 levels across PAH subtypes. They found significantly elevated levels in patients with CTD-PAH and IPAH, with strong associations with disease severity, clinical worsening, and reduced survival [99]. Elevated IGFBP4 levels were strongly associated with increased PAH severity, poorer survival, and disease progression [89, 90].

In a broader proteomic screen, Rhodes et al. utilized SOMAscan V3 to analyze 1129 plasma proteins across multiple PAH cohorts. They identified nine proteins (**IL1R4**,** EPO**,** Factor D**,** IGFBP1**,** TIMP2**,** TIMP1**,** Factor H**,** Plasminogen**, and **EPO**) that independently predicted survival beyond NT-proBNP [91]. Later, using an expanded SOMAscan V4 (> 4000 proteins), they identified 31 proteins significantly associated with prognosis, independent of 6MWD, age, sex, or NT-proBNP. From this, they derived a six-marker panel (**SVEP1**,** PXDN**,** renin**,** NRP1**,** TSP2**,** PRDX4**) that improved risk stratification when integrated with clinical variables [92].

Harbaum et al. also leveraged SOMAscan (V4) to analyze the plasma proteome in idiopathic and heritable PAH. Among 4152 annotated proteins, 208 were differentially expressed between PAH patients and healthy controls. Of these, 49 proteins, including established markers such as BNP and ANG2, were predictive of long-term survival. Integration with protein quantitative trait loci (pQTL) and Mendelian randomization (MR) analyses identified two proteins, **NET4** (netrin-4, encoded by NTN4) and **TSP2** (thrombospondin-2, encoded by THBS2), as having a causal role in PAH [93].

Further work by Bouzina et al. employed the Olink assay to measure plasma metabolic biomarkers in 48 treatment-naïve PAH patients at diagnosis, with 33 of them re-evaluated during early follow-up, alongside 16 healthy controls. Among the analytes, **fibroblast growth factor-23 (FGF-23)** was found to be significantly elevated in PAH patients and showed strong associations with disease severity, adverse hemodynamics, and higher risk stratification profiles. Importantly, FGF-23 levels decreased following treatment and correlated with improvements in clinical parameters. These findings highlight FGF-23 as a promising biomarker for disease monitoring and therapeutic response evaluation in PAH, warranting validation in larger, longitudinal cohorts [94].

Inflammatory proteomics has also yielded promising results. Sweatt and colleagues identified a circulating proteomic panel of 48 cytokines, chemokines, and factors using multiplex immunoassay. These cytokine profiles distinguish PAH immune phenotypes with differing clinical risks, independent of World Health Organization group 1 subtypes [108]. Mickael and colleagues utilized the Olink inflammation marker panel to simultaneously analyze 92 biomarkers in the peripheral blood of patients with clinically characterized IPAH and patients with scleroderma-associated pulmonary arterial hypertension (SSc-PAH), identifying 20 differentially expressed markers. Among these, six markers (**AXIN-1**, **ST1A1**, **CXCL1**, **STAMBP**, **SIRT2**, and **CASP8**) were increased only in IPAH, while 11 inflammatory markers were augmented in patients with SSc-PAH and IPAH: **VEGFA**, **CD8A**, **CXCL9**, **MMP1**, **IL-15RA**, **PD-L1**, **HGF**, **TNF**, **X4EBP1**,** CCL28**, and **CD40** [95].

Yokokawa et al. further focused on inflammation-related proteins using the Olink platform and found that elevated plasma levels of **FSTL3** and **SPON1** were independently associated with death or lung transplantation at the time of PAH diagnosis with incremental prognostic value on top of the 2015 ESC/ERS, the REVEAL 2.0 risk scores and the refined 4-strata risk assessment [96]. The attractiveness of these two proteins as new biomarkers in PAH is supported by findings showing similar results in independent cohorts with different assessment methods [63, 92].

Several early-phase proteomic studies, though limited by small sample sizes and early-generation techniques, have contributed to the identification of potential novel biomarkers in PAH. In adult IPAH patients, Abdul-Salam et al. employed SELDI-TOF MS to screen for differentially expressed proteins in plasma samples from 27 patients and 26 controls, detecting 234 unique protein ions. Among these, **complement 4a (C4a des Arg)** was confirmed via ELISA to be elevated in IPAH, achieving 92% classification accuracy at a cut-off of 0.6 µg/mL. However, broader validation is still needed [97].

Similarly, Zhang et al. utilized 2-DE and matrix-assisted laser desorption/ionization time-of-flight mass spectrometry (MALDI-TOF-MS) for clinical screening of serum proteins in 10 IPAH patients compared to 10 normal subjects, identifying nine proteins and their isoforms that were significantly different between groups. **Leucine-rich α-2-glycoprotein (LRG)** concentrations were confirmed by ELISA to be 2.7-fold higher in IPAH patients compared to controls and correlated significantly with right ventricular end-diastolic and right atrial diameters [98].

In a separate study focusing on pediatric patients with congenital ventricular septal defects, iTRAQ-based proteomics identified 107 differentially expressed plasma proteins between those with and without PAH. Notably, elevated levels of **adiponectin (ADIPO)**, **dopamine β-hydroxylase (DBH)**, and **alanyl membrane aminopeptidase (ANPEP)** were validated in an independent cohort. A composite risk score derived from these markers achieved an AUC of 0.87 in predicting PAH onset, suggesting potential utility in early stratification of children with congenital heart disease [99].

### Challenges and future directions

The discovery of PAH biomarkers has advanced significantly, yet several challenges remain that hinder their translation into clinical practice. Most emerging biomarkers are still in the experimental and basic research stages, lacking the large-scale clinical validation required for their widespread adoption. A major limitation of existing biomarkers is their insufficient specificity. While some biomarkers demonstrate high predictive and prognostic value across a range of diseases, such as left heart failure and pulmonary embolism, their lack of specificity makes them suboptimal for the precise diagnosis of PAH. However, these biomarkers hold great promise as prognostic indicators, providing valuable insights into patient health and disease progression. For example, they can be used to predict long-term outcomes, guide disease management, and stratify patients by risk. To establish their clinical utility, large-scale validation studies with well-defined patient cohorts are essential. Population-based case-control studies are particularly important to evaluate the prognostic performance of these biomarkers compared to established standards.

Another challenge lies in the reliance on mass spectrometry techniques for biomarker discovery. Although effective, the biomarkers identified using specific technical platforms or cohorts often lack generalizability. Standardization of sample collection, processing, and analysis protocols is critical to ensure reproducibility and comparability across studies. Variations in pre-analytical and analytical procedures can lead to inconsistent results, which impede the validation and clinical implementation of potential biomarkers. The antibody-based Olink and the aptamer-based SOMAscan allowed used for particular biomarker or biomarker panels. In a detection panel, different proteins separately reflect different pathological conditions in PAH, so that provides comprehensive information for prognosis including inflammation, pulmonary vascular remodeling, cardiac hypertrophy and so on. Such advances could significantly improve the accuracy of diagnostic and prognostic assessments and enhance clinical therapeutic strategy.

The ongoing development of novel detection technologies and analytical methodologies presents opportunities to expand the clinical application of protein biomarkers. As research and clinical data continue to accumulate, these findings will lay the groundwork for identifying effective biomarkers. However, it remains critical to integrate and update these findings efficiently. Open data sharing, cross-center collaboration, and the creation of technical exchange platforms will be key to advancing this field. Such initiatives will require substantial investment in resources and the coordinated efforts of international organizations. Collaboration among researchers, clinicians, and regulatory bodies is vital for the development of guidelines and standard operating procedures to incorporate biomarkers into routine diagnostic and prognostic protocols. This collaborative approach will ensure the successful integration of protein biomarkers into clinical practice, ultimately improving PAH diagnosis, treatment, and patient outcomes.

An additional future challenge is the integration of novel biomarkers into existing clinical algorithms. Current diagnostic workflows for PAH rely on hemodynamic assessment, imaging, and functional testing, with NT-proBNP/BNP as the main circulating markers. Newly identified protein biomarkers could complement these tools at multiple levels. In screening, markers such as apelin, endothelin-1, or osteopontin may help flag high-risk patients earlier and reduce diagnostic delay. In prognosis, emerging biomarkers and multi-protein panels identified by Olink and SOMAscan could be incorporated into validated multiparametric scores such as the REVEAL 2.0 calculator or ESC/ERS risk stratification model, thereby improving precision in identifying intermediate-risk patients and guiding timely therapy escalation. These integrative approaches hold promise to bridge the gap between discovery and clinical application, but require prospective validation in large, diverse cohorts.

## Conclusion

Proteomic advances have vastly expanded our armamentarium of circulating biomarkers, illuminating new facets of PAH pathobiology—from myocardial strain and vascular remodeling to inflammation, fibrosis and end-organ dysfunction. While classical markers such as BNP and NT-proBNP remain foundational, high-throughput platforms (Olink, SOMAscan, DDA-MS) have uncovered panels and individual proteins (LTBP-2, IGFBP family members, NET4/TSP2, FGF-23, inflammatory cytokines) with promising diagnostic and prognostic performance.

Realizing their full potential will require rigorous standardization, large-scale validation across PAH phenotypes, and integration with clinical, imaging and genetic data. By addressing these challenges through collaborative, multi-center efforts and embracing emerging computational tools, the next generation of biomarkers may enable truly personalized care-earlier diagnosis, precise risk stratification, dynamic treatment monitoring, and ultimately, improved outcomes for patients with pulmonary arterial hypertension.

## Data Availability

Not applicable.

## Abbreviations

PH: Pulmonary hypertension
RV: Right ventricle
PAH: Pulmonary arterial hypertension
LC-MS: Liquid chromatography-mass spectrometry
HPLC: High-performance liquid chromatography
DDA-MS: Data-dependent acquisition mass spectrometry
MALDI-TOF-MS: Matrix-assisted laser desorption/ionization time-of-flight mass spectrometry
mPAP: mean pulmonary artery pressure
RAP: Right atrial pressure
PVR: Pulmonary vascular resistance
CO: Cardiac output
CI: Cardiac index
6MWD: 6-Minute Walk Distance
WHO FC: World Health Organization Functional Classification
NYHA: New York Heart Association
ECM: Extracellular matrix
IPAH: Idiopathic PAH
CHD-PAH: Congenital heart disease-associated PAH
CTD-PAH: Connective tissue disease-associated PAH
BNP: Brain natriuretic peptide
NT-proBNP: N-terminal pro-BNP
cTnI/cTnT: Cardiac troponins I and T
ET-1: Endothelin-1
APLNR: Apelin receptor
ES: Endostatin
FSTL3: Follistatin-like 3
HDGF: Hepatoma-derived growth factor
CTHRC1: Collagen triple helix repeat-containing protein 1
sST2: Soluble ST2
CRP: C-reactive protein
SCr: Creatinine
CysC: Cystatin C
CA2: Anhydrase 2
KLKB1: Kallikrein
IGFBP: Insulin-like growth factor-binding protein
pQTL: Protein quantitative trait loci
FGF-23: Fibroblast growth factor-23
LRG: Leucine-rich α-2-glycoprotein
ADIPO: Adiponectin
DBH: Dopamine β-hydroxylase
ANPEP: Alanyl membrane aminopeptidase
SvO_2_: Venous oxygen saturation
